# Prognostic value of programmed cell death protein 1 expression on CD8+ T lymphocytes in pancreatic cancer

**DOI:** 10.1038/s41598-017-08479-9

**Published:** 2017-08-10

**Authors:** Tao Shen, Liangjing Zhou, Hua Shen, Chengfei Shi, Shengnan Jia, Guo Ping Ding, Liping Cao

**Affiliations:** Department of General Surgery, Sir Run Run Shaw Hospital, School of Medicine, Zhejiang University, Hangzhou, 310000 China

## Abstract

Pancreatic cancer is one of the most aggressive malignancies and has a highly immunosuppressive tumour microenvironment. Immune checkpoint blockade has led to remarkable and durable objective responses in a number of malignancies and antibody-based strategies targeting programmed cell death protein 1 (PD-1) are showing promise where traditional modalities of surgery, radiotherapy, and chemotherapy have failed. In this study, we examined the clinical value of PD-1 protein expression by CD8+ peripheral T lymphocytes or tumour-infiltrating T lymphocytes (TILs) in pancreatic ductal adenocarcinoma (PDAC). Expression of PD-1 protein on CD8+ TILs correlated with overall survival and clinicopathological characteristics such as clinical stage, N classification, and M classification. Similar findings were observed for the expression of PD-1 protein on peripheral CD8+ T cells, whereas its expression on peripheral CD4+ T cells showed no significance. Comparison of the levels of PD-1 protein expressed by peripheral CD8+ T cells before and 4 weeks after surgery indicated that preoperative and postoperative status of peripheral PD-1 expression was unchanged. Our findings showed that PD-1 protein expressed by peripheral or tumour-infiltrated CD8+ T cells was a promising biomarker for diagnosis and prognosis in PDAC and might help guide future immunotherapies.

## Introduction

Pancreatic cancer (PC) is currently the fourth leading cause of cancer death, with a 5-year survival rate of 7–8%^1^. Estimated numbers of new cases of pancreatic cancer and deaths are estimated to be 53,070 and 41,780 respectively in the United States in 2016, based on the increased incidence and death rate between 1992 and 2012^1^. This extremely poor prognosis is in part because 53% of cases are diagnosed at an advanced stage due to a lack of efficient methods for early detection^2^. Surgical resection is the mainstay of therapy; however, the radical resection rate is only approximately 18% because of the high incidence of invasion and metastasis^3^. Moreover, even for patients who undergo a radical resection, prognosis remains poor^4^. Radiotherapy and chemotherapy regimens that have been routinely applied for postoperative treatment have shown limited overall effectiveness for PC at a metastatic stage^5^. Therefore, a deeper exploration of the molecular pathogenesis of PC metastasis and identification of novel therapies against PC are two issues of crucial importance.

Pancreatic cancer is characterised by insidious early symptoms, rapid progression, and poor prognosis^6^, associated with mechanisms of immune evasion^7^. Previous studies suggest that immune suppressors play a key role in enabling malignant tumours to evade immune surveillance^8, 9^. Metastatic cancer cells escape from immune surveillance with the help of an immune checkpoint, which represents a characteristic inhibitor of the antitumour immune response^10, 11^. The immune checkpoint involves a series of immunosuppressive molecules associated with downregulation of immune responses to protect against an autoimmune response and maintain peripheral autotolerance^12^.

As a crucial checkpoint in the immunosuppressive pathway, the programmed cell death protein 1 (PD-1) receptor is upregulated in T cells following interaction with tumour antigens^13^. When bound to its ligands, such as programmed death-ligand 1 (PD-L1), the PD-1 receptor has been shown to limit the antitumour activity of T cells, resulting in promotion of immunosuppression and further facilitating tumour growth and progression^14^. It has been reported that high expression of PD-1 on CD8+ T cells is associated with poor prognosis in renal cell carcinoma and Hodgkin lymphoma^15, 16^. However, PD-1 expression does not always indicate a poor prognosis. In some tumours, such as human papilloma virus (HPV)-associated head and neck cancer, follicular lymphoma, and colorectal cancer, infiltration by PD-1+ T cells is associated with good prognosis^17–19^. Even though the prognostic significance of PD-1 expression is different for different tumour histologies, antibody therapy targeting PD-1 has emerged as a promising approach for tumour immunotherapy^20, 21^. However, the success of anti-PD-1 therapy depends on a high mutation burden and the presence of neoantigen-specific CD8+ T cells^22^. The expression of PD-1 protein and its clinicopathological and prognostic significance in pancreatic ductal adenocarcinoma (PDAC) remain unclear.

To determine whether PD-1 protein is a suitable target for PDAC immunotherapy we measured the level of PD-1 expression on tumour-infiltrating CD8+ T lymphocytes by immunohistofluorescence and on peripheral T lymphocytes by flow cytometry in healthy donors and patients with intraductal papillary mucinous neoplasm (IPMN) or PDAC. To explore whether peripheral PD-1 expression in PDAC patients who undergo surgical resection is a suitable monitoring indicator for postoperative immunotherapy targeting PD-1 protein, we also evaluated the changes of PD-1 expression on peripheral T lymphocytes at 4 weeks after curative surgical resection. Finally, we found that PD-1 protein expressed by peripheral or tumour-infiltrated CD8+ T cells was a promising biomarker for diagnosis and prognosis in PDAC and might help guide future immunotherapies.

## Results

### PD-1 and CD8 expression on tumour-infiltrating lymphocytes

**Figure 1:**
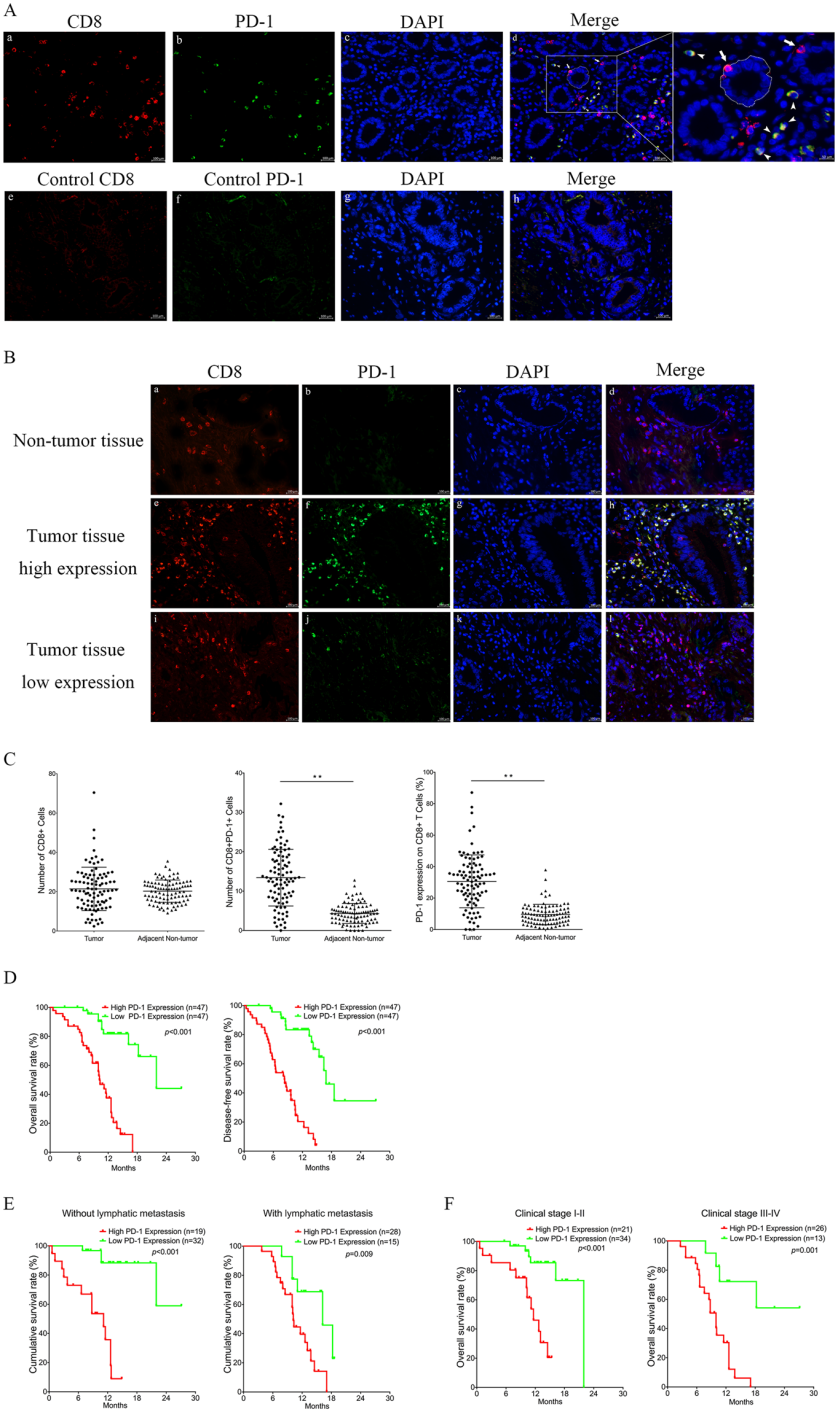
Level of PD-1 expression on CD8+ T cells in PDAC tissue samples and its correlation with overall survival rate in pancreatic ductal adenocarcinoma patients. (**A**) Tissues derived from surgical resection of PDAC patients were stained with antibodies to human CD8 (red) (a) and PD-1 (green) (b) and counterstained with DAPI (blue) (c). Isotype control antibodies for anti-PD-1 and anti-CD8 were included in each experiment (e, f). The merged graph (d) shows that the pancreatic duct (dotted line) was mainly infiltrated with CD8+ T cells (arrows) whereas stroma was mainly infiltrated with PD-1+ CD8+ T cells (arrowheads) (original magnification, ×400). A representative experiment is shown in B. Quantitative measurement of CD8+ and PD-1+ CD8+ Tumour-infiltrating lymphocytes (TILs) was performed in at least four random high power fields (×400), and the expression level of PD-1 on CD8+ TILs was calculated as follows: expression level, % = (PD-1+ CD8+ TILs/CD8+ TILs) × 100. For quantitative variables, the median was used as a cut-off. Mean (±SD) number of infiltrated PD-1+ CD8+ T cells and CD8+ T cells and mean (±SD) expression of PD-1 protein on tumour-infiltrating CD8+ T cells from 94 paired samples of PDAC tumour tissues and adjacent non-tumour tissues is shown in C (paired-samples T-test, **P < 0.001). For the analysis of overall survival or disease-free survival, high and low levels were defined using the median level of PD-1 expression on tumour-infiltrated CD8+ T cells as the cut-off value. Survival analysis was performed using the Kaplan–Meier method and log-rank test. (**D**) High expression level of PD-1 on CD8+ T cells in tumour tissue was significantly correlated with poor survival of PDAC patients. Correlation between PD-1 expression and overall survival rate in PDAC patients was independent of clinical stage (**E**) and status of lymphatic metastasis (**F**).

We examined the expression of PD-1 and CD8 in tumour-infiltrating lymphocytes by double immunofluorescence staining (IF) of 94 pairs of resection specimens (tumour tissues and matched adjacent non-tumour tissues) from PDAC patients (34 female and 60 male patients with a median age of 65 years; age range, 43–88 years). PD-1 protein was expressed on the membrane of CD8+ T lymphocytes, which were mainly infiltrated in the pancreatic stroma (Fig. 1A). The number of CD8+ PD-1+ cells (mean ± SD) was 13.42 ± 7.20 in tumour tissues and 4.33 ± 2.55 in adjacent non-tumour tissues (Fig. 1C; P < 0.001). This indicated that adjacent non-tumour tissues were less infiltrated by PD-1+ CD8+ T lymphocytes than tumour tissues (Fig. 1B). The number of CD8+ T cells (mean ± SD) was 21.42 ± 11.06 in tumour tissues and 20.28 ± 5.64 in adjacent non-tumour tissues (Fig. 1C; P = 0.375). There was no significant difference in CD8+ T lymphocyte infiltration between tumour tissues and adjacent non-tumour tissues. More importantly, the level of PD-1 expression on CD8 + cells (mean ± SD %) was 30.62 ± 16.80% in tumour tissues and 9.35 ± 6.60% in adjacent non-tumour tissues (Fig. 1C; P < 0.001). Increased expression of PD-1 on tumour-infiltrating CD8+ T lymphocytes was observed in 82.98% (78/94) of tumour tissues. These data showed that the level of PD-1 expression on CD8+ T lymphocytes was higher in tumour tissue than in adjacent non-tumour tissue (Fig. 1B).

### The clinicopathological significance of PD-1 expression on tumour-infiltrating CD8+ T lymphocytes in PDAC

To determine the significance of PD-1 expression on tumour-infiltrating CD8+ T lymphocytes in PDAC, a retrospective analysis was performed. The Chi-square test was used to assess correlations between PD-1 expression and clinicopathological parameters (including age, gender, tumour size, tumour site, T classification, N classification, M classification, clinical stage, and histological stage). As shown in Table 1, PD-1 expression on tumour-infiltrating CD8+ T lymphocytes in PDAC was significantly correlated with clinical stage (χ^2^ = 6.136, P = 0.013), N classification (χ^2^ = 7.575, P = 0.023), and M classification (χ^2^ = 17.375, P < 0.001). No significant correlations were observed between PD-1 expression level and age, gender, tumour size, tumour site, T classification, or histological stage. These results indicated that increased expression of PD-1 protein on tumour-infiltrating CD8+ T lymphocytes might play an important role in immune escape and promoting metastasis in patients with PDAC.

**Table 1:** Correlation of PD-1 expression on tumour-infiltrating CD8+ T cells with major clinicopathological parameters in patients with pancreatic ductal adenocarcinoma (PDAC). The bold numbers represent significant differences with P < 0.05.

Clinicopathological parameters	n	PD-1 expression		χ2 value	*P* value
		Low	High		
**Age (years)**					
<65	44	20	24	0.684	0.408
≥65	50	27	23		
**Gender**					
Male	60	29	31	0.184	0.668
Female	34	18	16		
**Tumor site**					
Head	73	38	35	0.552	0.458
Body+ Tail	21	9	12		
**Histological grade**					
Well	33	13	20	2.288	0.130
Poor + Moderate	61	34	27		
**Tumor size (cm)**					
≤3.0	52	25	27	0.172	0.678
>3.0	42	22	20		
**Tumor classification**					
T1~T2	71	35	36	0.058	0.810
T3~T4	23	12	11		
**Node classification**					
N0	51	32	19	**7.575**	**0.023**
N1	29	11	18		
N2	14	4	10		
**Distant metastasis**					
M0	63	41	22	**17.375**	**<0.001**
M1	31	6	25		
**Clinical stage (AJCC)**					
I~II	55	34	21	**6.136**	**0.013**
III~IV	39	13	26		

### Prognostic significance of PD-1 expression on tumour-infiltrating CD8+ T lymphocytes in PDAC patients

To determine the prognostic value of PD-1 expression level in PDAC, we used the Kaplan–Meier method and log-rank test to analyse the relationship between PD-1 expression and clinical follow-up information. The patients were divided into high/low expression groups according to the median value. As shown in Fig. 1D, high PD-1 expression was associated with short overall survival (χ^2^ = 35.42, P < 0.001) and short disease-free survival (χ^2^ = 40.39, P < 0.001), suggesting that high expression of PD-1 on tumour-infiltrating CD8+ T cells was associated with poor prognosis. In addition, we determined the correlation between PD-1 expression and overall survival in PDAC patients with clinical stage I-II *v*ersus III-IV and in patients with versus without lymphatic metastasis. Analysis by the Kaplan–Meier method and log-rank test showed that higher PD-1 expression correlated with shorter overall survival independent of clinical stage (Fig. 1E) and N stage (Fig. 1F). Furthermore, a multivariate survival analysis by Cox proportional hazard model confirmed PD-1 expression, T stage, M stage and Clinical stage as independent predictors of the overall survival in 94 PDAC patients with R0 resection (Table 2). These results further indicated that increased expression of PD-1 on tumour-infiltrating CD8+ T lymphocytes was an adverse factor in PDAC, and might be a novel predictor for prognosis in patients with PDAC.

**Table 2:** Multivariate Cox regression analysis of prognostic parameters for survival in patients with pancreatic ductal adenocarcinoma (PDAC). HR: Hazard ratio; CI: Confidence interval. The bold numbers indicate significant differences with P < 0.05.

Prognostic parameter	Multivariate analysis		
	HR	95% CI	P value
**PD-1 expression**			
(High vs. Low)	6.002	2.248–16.022	**0.001**
**Age**			
(<65 vs. ≥ 65)	0.993	0.950–1.038	0.768
**Gender**			
(Male vs. Female)	1.578	0.718–3.467	0.256
**Tumor site**			
(Body + Tail vs. Head)	0.551	0.225–1.348	0.192
**Histological grade**			
(Well vs. Poor + Moderate)	0.954	0.438–2.079	0.907
**Tumor size**			
(≤3.0 vs. >3.0)	0.767	0.329–1.788	0.539
**Tumor classification**			**0.022**
T2 vs. T1	2.280	0.709–7.330	0.166
T3 vs. T1	1.805	0.285–11.494	0.530
T4 vs. T1	6.623	1.109–39.370	**0.038**
**Node classification**			0.069
N1 vs. N0	3.021	0.837–10.989	0.091
N2 vs. N0	2.888	0.772–10.801	0.115
**Metastasis classification**			
(M1 vs. M0)	4.917	1.456–16.611	**0.010**
**Clinical stage**			0.120
II vs. I	5.408	1.058–27.634	**0.043**
III vs. I	2.371	0.352–15.985	0.375
IV vs. I	4.917	1.456–16.611	**0.010**

### Expression of PD-1 protein on peripheral T lymphocytes and its value as a biomarker in PDAC

**Figure 2:**
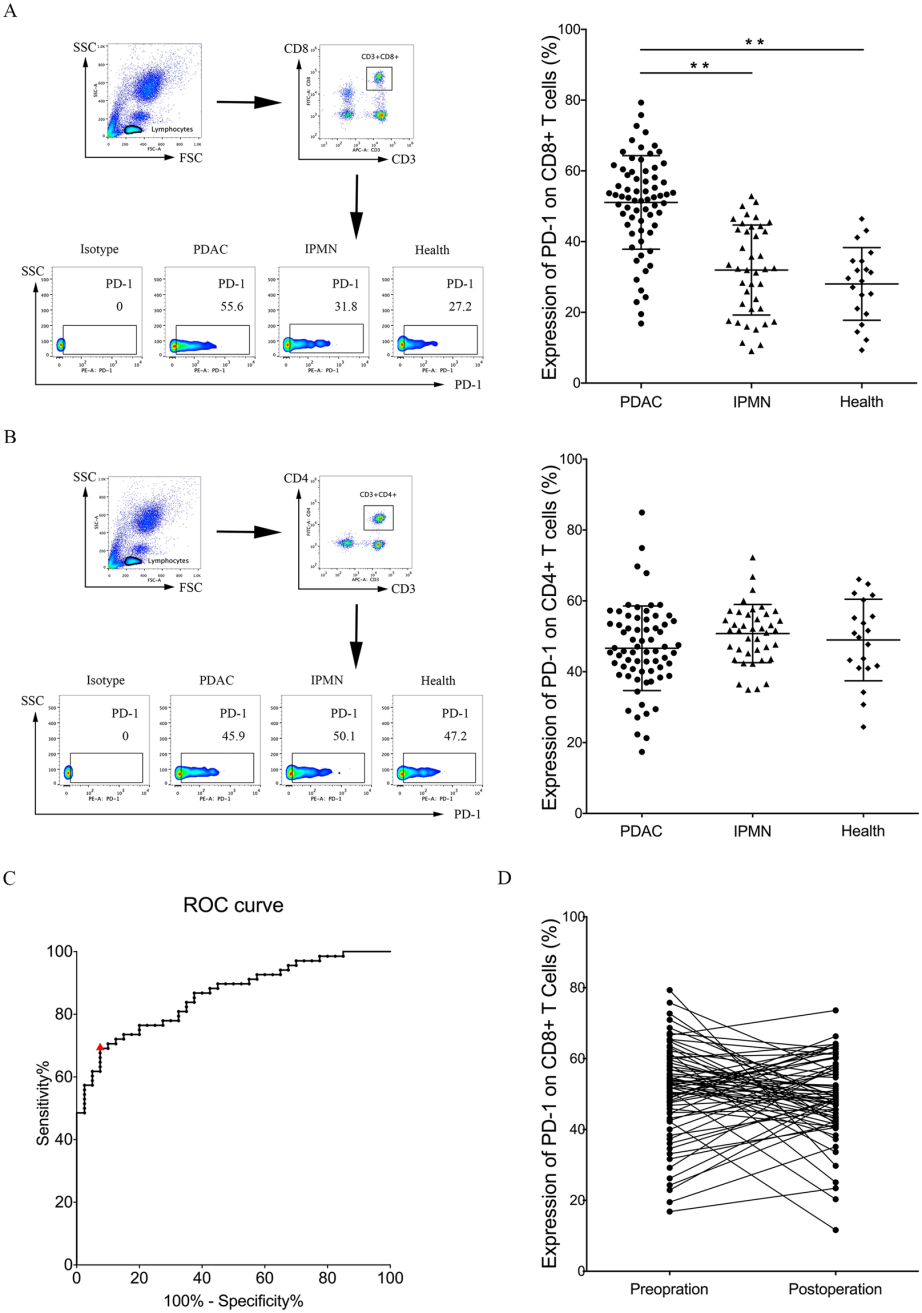
PD-1 protein expression is increased on peripheral CD8+ T cells but not on peripheral CD4+ T cells in patients with pancreatic ductal adenocarcinoma. The levels of PD-1 protein expressed by peripheral CD8+ T lymphocytes (**A**) and peripheral CD4+ T lymphocytes (**B**) in 68 patients with PDAC, 40 patients with intraductal papillary mucinous neoplasm (IPMN), and 20 healthy donors were detected by flow cytometry. Graphs show quantification of FACS data. Flow cytometry pseudo colour of lymphocytes and CD3+ CD8+ (A) or CD3+ CD4+ (**B**) cells and representative smoothing pseudo colour of PD-1+ cells are displayed. Data shown are mean ± standard deviation, **P < 0.001. (**C**) ROC curve for the correlation of PD-1 expression on peripheral CD8+ T cells with PDAC. Area under the curve is 0.8607 (95% confidence interval 0.7938 to 0.9276), P < 0.001. The cut-off value of >47.81% PD-1 expression on CD8+ T cells is designated by the red triangle. (**D**) There was no significant difference in the level of PD-1 expression between preoperative and 4-week postoperative peripheral CD8+ T lymphocytes. Wilcoxon matched-pairs signed rank test; P = 0.498.

Recent studies indicated that higher expression of PD-1 on peripheral CD4+ T cells was associated with poor prognosis in patients with chronic lymphocytic leukaemia or diffuse large B-cell lymphoma^23, 24^. However, there were few reports on peripheral PD-1 expression in pancreatic cancer. Here, the expression levels of PD-1 on peripheral T lymphocytes were detected using flow cytometry in128 patients including 68 newly diagnosed PDAC patients(31 female and 37 male patients with a median age of 60 years; age range, 38–78 years), 40 patients with IPMN(17 female and 23 male patients with a median age of 63 years; age range, 41–82 years) and 20 healthy donors (10 female and 10 male patients with a median age of 62 years; age range, 40–79 years). In this study, the positive rate for PD-1 expression on peripheral CD8+ T lymphocytes was 5% (1/20) in healthy donors, 27.5% (11/40) in IPMN patients, and 76.47% (52/68) in PDAC patients (using the median as the cut-off). PD-1 was expressed at a significantly higher level on peripheral CD8+ T lymphocytes in PDAC than that in IPMN (51.08 ± 13.22 vs. 31.96 ± 12.72; P < 0.001) and in healthy donors (51.08 ± 13.22 vs. 28.04 ± 10.27; P < 0.001) (Fig. 2A), whereas there was no significant difference in the expression of PD-1 on CD4+ T lymphocytes among the three groups (Fig. 2B). In addition, in comparison of IPMN patients with healthy donors, there was no significant difference in PD-1 expression on CD4+ (Fig. 2B) or CD8+ T lymphocytes (Fig. 2A). These data of PD-1 expressions on peripheral CD4+ or CD8+ T lymphocytes among three groups are shown in Supplementary Table S1.

The power of PD-1 expression on peripheral CD8+ T cells to discriminate PDAC from IPMN was analysed using receiver operator characteristic (ROC) curves. There was high concordance between PD-1 expression on peripheral CD8+ T cells and PDAC, with an area under the ROC curve (AUC) of 86.07% (95% confidence interval [CI] 0.7938 to 0.9276, P < 0.001) (Fig. 2C), which indicated a moderate discriminatory power. Patients with PD-1 expression > 47.81%, the cutoff point that combined maximal sensitivity with best specificity, had a likelihood ratio of 9.22; the sensitivity and specificity of PDAC using this cutoff point was 69.12% (95% CI 56.74 to 79.76%) and 92.5% (95% CI 79.61 to 98.43%), respectively. The ROC curve demonstrated that the level of PD-1 expression on peripheral CD8+ T lymphocytes was a good biomarker to distinguish between patients with PDAC and those with IPMN.

### Clinicopathological significance of PD-1 expression on peripheral CD8+ T lymphocytes in PDAC

To evaluate the clinical significance of PD-1 expression on peripheral CD8+ T lymphocytes in PDAC, we examined the correlation between PD-1 expression level and major clinicopathological parameters (age, gender, tumour size, tumour site, T classification, N classification, M classification, clinical stage, and histological stage). As shown in Table 3, PD-1 expression on peripheral CD8+ T lymphocytes of PDAC patients was closely correlated with N classification (χ^2^ = 19.127, P < 0.001), M classification (χ^2^ = 4.121, P = 0.042), and clinical stage (χ^2^ = 7.173, P = 0.007). No significant correlations were observed between PD-1 expression level and age, gender, tumour size, tumour site, T classification, or histological stage. The same results were obtained for PD-1 expression on tumour-infiltrating CD8+ T lymphocytes. Together, these findings demonstrate that the level of PD-1 expression on CD8+ T lymphocytes, either peripheral or tumour-infiltrating lymphocytes, might be an important biomarker of diagnosis and prognosis for patients with PDAC.

**Table 3:** Correlation of PD-1 expression on peripheral CD8+ T cells with major clinicopathological parameters in patients with pancreatic ductal adenocarcinoma (PDAC).

Clinicopathological parameters	n	PD-1 expression		χ2 value	*P* value
		Low	High		
**Age (years)**					
<60	23	10	13	0.464	0.496
≥60	45	24	21		
**Gender**					
Male	37	21	16	1.482	0.223
Female	31	13	18		
**Tumor site**					
Head	38	18	20	0.239	0.625
Body + Tail	30	16	14		
**Histological grade**					
Well	29	17	12	1.503	0.220
Poor + Moderate	39	17	22		
**Tumor size (cm)**					
≤3.0	23	14	9	1.643	0.200
>3.0	45	20	25		
**Tumor classification**					
T1~T2	50	23	27	1.209	0.272
T3~T4	18	11	7		
**Node classification**					
N0	36	27	9	**19.127**	**<0.001**
N1	18	4	14		
N2	14	3	11		
**Distant metastasis**					
M0	44	26	18	**4.121**	**0.042**
M1	24	8	16		
**Clinical stage (AJCC)**					
I~II	37	24	13	**7.173**	**0.007**
III~IV	31	10	21		

The bold numbers indicate significant differences with P < 0.05.

### PD-1 expression on peripheral CD8+ T lymphocytes is a suitable indicator for immunotherapy targeting PD-1 blockage

Above analyses about preoperative PD-1 expression on peripheral CD8+ T lymphocytes indicate that preoperative peripheral PD-1 expression was a potential biomarker for PDAC immunotherapy. However, it was unknown whether the expression of PD-1 on peripheral CD8+ T lymphocytes would be changed after surgical resection in PDAC patients. And it was still undiscovered whether postoperative peripheral PD-1 expression was a suitable indicator to help guide postoperative immunotherapy. To this end, 4-week postoperative peripheral CD8+ T lymphocytes of the above 68 PDAC patients were collected and analysed by flow cytometry to determine changes in PD-1 expression. Wilcoxon matched-pairs signed rank test demonstrated no significant differences in the level of PD-1 expression between preoperative and postoperative peripheral CD8+ T lymphocytes (Fig. 2D; P = 0.498). This result indicated that preoperative and postoperative status of peripheral PD-1 expression was unchanged, and the peripheral PD-1 expression was sustained in PDAC patients. Whether preoperative or postoperative PD-1 expression on peripheral CD8+ T lymphocytes, they all presented guiding significance for PDAC immunotherapy.

## Discussion

As a member of the CD28 immunoglobulin superfamily, PD-1 is a 50- to 55-kDa type I transmembrane protein that was first detected on the surface of apoptotic cells in 1992^25, 26^. Its expression has since been widely found on T cells, B cells, myeloid-derived cells^27^, and a subset of thymocytes^28^. Binding of PD-1 to its ligands PD-L1 and/or PD-L2 transmits a negative signal through the immunoreceptor tyrosine-based switch motif (ITSM)^29^, which negatively regulates cytokine secretion and promotes T cells into apoptosis or exhaustion^30, 31^. The capability for antitumour immune responses is accordingly suppressed^32^. PD-1 overexpression has been detected in a variety of different human malignancies including melanoma, non-small cell lung cancer, and renal cell carcinoma, in which it predicts a poor prognosis^33–35^. In contrast, in HPV-associated head and neck cancer, follicular lymphoma, and colorectal cancer, infiltration by PD-1+ T cells is associated with good prognosis^17–19^. Wang *et al*. confirmed that combined PD-1/PD-L1 expression, but not PD-L2, could serve as an independent prognostic marker in PDAC^36^. However, PD-L1 expression on pancreatic cancer cells is sparse^37^. In the first multicenter phase 1 trial of anti-PD-L1 therapy, a total of 207 patients, including 14 patients with pancreatic cancer, had received anti-PD-L1 antibody. But none of the 14 PDAC patients had objective responses (NCT00729664)^38^. The anti-PD-1 mAb pembrolizumab is currently tested ranging from neoadjuvant to combination with chemotherapy (NCT02305186, NCT02362048, NCT02268825, NCT02054806) or targeted drugs (NCT02452424, NCT02362048, NCT02452424). And the pidilizumab (CT-011), another anti-PD-1 mAb, is now tested as adjuvant therapy after resection of PDAC (NCT01313416)^39^. Immunotherapy targeting anti-PD-1 appeared to be more promising than targeting anti-PD-L1. To date, however, there is a lack of systematic research on the immune checkpoint of PD-1 in PDAC. The level of PD-1 expression and its role in the development of PDAC is also unclear.

In this study, we first demonstrated that PD-1 expression by infiltrated CD8+ T cells was increased in PDAC tissues compared with adjacent non-tumour tissues at the protein level. We also showed significant correlations between PD-1 expression and certain clinicopathological features, including clinical stage, N classification, and M classification. Moreover, the level of PD-1 expression on TILs inversely correlated with postoperative survival. Interestingly, stratified analysis showed that tumour-infiltrating PD-1 expression was a risk factor for poor prognosis of PDAC patients independent of clinical stage and N classification. Furthermore, multivariate analysis confirmed the possibility that PD-1 protein could be useful as an independent risk factor for poor prognosis in the 94 PDAC patients with R0 resection. A recent study showed that PD-L1–positive patients had a poorer prognosis than PD-L1–negative patients^40^. Our experiment confirmed the prognostic significance of PD-1 protein level, and further validated the hypothesis that the PD-1/PD-L1 pathway played a negative role in tumour immunity in pancreatic cancer.

More importantly, we measured the level of PD-1 protein expressed on peripheral CD8+ T cells by flow cytometry. Rosenberg’s research revealed that the tumour antigen specificities and T-cell receptor repertoires of circulating and tumour-infiltrating CD8+ PD-1+, but not CD8+ PD-1−, cells appeared similar, implying that the circulating CD8+ PD-1+ lymphocytes might provide a view into the tumour-resident antitumour lymphocytes^41^. On this basis, we aimed to explore whether peripheral PD-1 status could be a novel diagnostic predictor for PDAC. Our study demonstrated that the expression of PD-1 on peripheral CD8+ T cells was markedly higher in PDAC patients than in IPMN patients or healthy donors. ROC analysis further confirmed the diagnostic significance of peripheral PD-1 expression for PDAC. Interestingly, high expression of PD-1 on peripheral CD8+ T cells was significantly correlated with clinical stage, N classification, and M classification of PDAC, consistent with the correlation between tumour-infiltrating PD-1 expression and clinicopathological features. Notably, measuring the level of PD-1 protein expressed by CD8+ T lymphocytes in peripheral blood is more convenient than measurement in surgical tissue samples and especially important in patients with unresected PDAC. Together, these results indicated that peripheral PD-1 expression might be a new predictor of diagnosis and prognosis for patients with PDAC.

Furthermore, we found that there was no obvious change in peripheral PD-1 expression after treatment by surgical resection. This finding indicated that, the level of PD-1 expression on peripheral CD8+ T lymphocytes was sustained and stable from pre-operation to 4-week after surgical operation. To date, surgery remains the only potential cure for pancreas cancer, but many patients who undergo surgical resection eventually undergo recurrence and die from the disease^4^. Therefore, it is imperative to develop neoadjuvant approaches for the prevention of recurrence after surgery. Previously, there is little research in exploring correlation between immune status and postoperative PDAC recurrence. We supposed that sustained overexpression of PD-1 on peripheral CD8+ T lymphocytes might be related to high rate of postoperative recurrence, and that postoperative immunotherapy targeting anti-PD-1 might improve the prognosis of PDAC patients. In addition, peripheral PD-1 expression on CD8+ T lymphocytes was a suitable monitoring indicator for immunotherapy after surgical operation.

In summary, our study demonstrated that high and refractory PD-1 expression on either peripheral or tumour-infiltrating T cells was associated with poor survival in PDAC patients. These data suggested that PD-1 expression might function as an important marker for diagnosis and prognosis and might be a potential molecular target for the treatment of PDAC. Especially, peripheral PD-1 expression was a suitable marker to help guide future immunotherapies targeting immune checkpoint inhibitors for postoperative patients or those with unresectable PDAC.

## Materials and methods

### Ethics Statement

The research protocol was reviewed and approved by the Research Ethics Committee of Sir Run Run Shaw Hospital, School of Medicine, Zhejiang University. All experiments were conducted in accordance with approved guidelines of the Sir Run Run Shaw Hospital, School of Medicine, Zhejiang University. All participants or their guardians provided written informed consent for scientific research statement.

### Patients

#### Tissue specimens

188 formalin-fixed and paraffin-embedded tissue specimens including 94 pancreatic ductal adenocarcinomas and 94 adjacent normal pancreatic tissues were obtained from 94 PDAC patients (34 female and 60 male patients with a median age of 65 years; age range, 43–88 years) who underwent R0 surgical resection at the Department of General Surgery, Sir Run Run Shaw Hospital of Zhejiang University School of Medicine between September 2012 and March 2014. All cases were confirmed by pathological diagnosis. Pathologic classification was performed according to 8th edition of AJCC. Among the 94 PDAC patients, 5 patients presented with stage IA disease, 21 patients presented with stage IB disease, 7 patients presented with stage IIA disease, 22 patients presented with stage IIB disease, 8 patients presented with stage III disease and 31 patients presented with stage IV disease. The cases of PDAC were selected in this study only if clinical data were available. For the analysis of disease-free survival or overall survival, the follow-up time was calculated from the date of surgery to the date of recurrence or death, or the last known follow-up, respectively. Recurrence included locoregional, local, and distant metastasis. None of them had received radiotherapy, chemotherapy, hormone therapy or other related anti-tumor therapies before or after surgery. For each case, tissue block containing tumor or adjacent normal pancreatic tissue was selected and 2–3 μm sections were cut.

#### Peripheral T lymphocytes samples

128 peripheral T lymphocytes samples were obtained from the peripheral blood of 108 pancreatic neoplasm patients including 68 PDAC patients (31 female and 37 male patients with a median age of 60 years; age range, 38–78 years) and 40 IPMN patients (17 female and 23 male patients with a median age of 63 years; age range, 41–82 years) and 20 healthy participants (10 female and 10 male patients with a median age of 62 years; age range, 40–79 years) at the Department of General Surgery, Sir Run Run Shaw Hospital of Zhejiang University School of Medicine between January 2015 and July 2016. All of the enrolled patients were newly diagnosed and underwent R0 surgical resection. All cases were confirmed by pathological diagnosis. Pathologic classification was performed according to 8th edition of AJCC. Among the 68 PDAC patients, 6 patients presented with stage IA disease, 15 patients presented with stage IB disease, 6 patients presented with stage IIA disease, 10 patients presented with stage IIB disease, 7 patients presented with stage III disease and 24 patients presented with stage IV disease. We excluded patients with an acute or chronic infection, inflammatory processes, a history of autoimmune disease, or those who received previous therapy with any corticosteroids or immunotherapy before the start of the treatment course. We also excluded patients who received radiotherapy, chemotherapy, hormone therapy or other related anti-tumor therapies within 4 weeks postoperatively.

### Immunofluorescence for PD-1 and CD8

Co-localization of CD8 and PD-1 was assessed by immunofluorescence staining in tissue samples. Paraffin-embedded tissue sections were dewaxed by dimethylbenzene and rehydrated through a gradient ethanol series. Then phosphate-buffered saline (PBS; pH 7.4) was used to wash the residual dimethylbenzene and ethanol. After they had been washed three times for 5 min, the sections were bathed on citric acid buffer (PH6.0) at 95–98 °C for 15 min for antigen retrieval. Naturally cooled to room temperature, the sections were incubated in 5% normal goat serum in PBS. Following the serum block, immunofluorescence staining of PD-1 and CD8 was carried out by co-incubation with mouse anti-PD-1 antibody (ab52587, Abcam) at dilution 1:200 and rabbit anti-CD8 antibody (ab4055, Abcam) at dilution 1:100 overnight and detected using goat anti-Mouse IgG H&L Alexa Fluor 488 (ab150117, Abcam) and goat anti-Rabbit IgG H&L Alexa Fluor 594 (ab150084, Abcam). Isotype-matched antibodies were used as negative controls. In each case, we checked that the secondary antibodies did not cross-react with the isotype or species of the other primary antibody immunoglobulin in the double immunofluorescence technique. Cell nuclei were stained with DAPI. Sections were digitally photographed using an Axiocam 506 color camera mounted on a Zeiss Observer.A1 microscope. Two authors (T.S. and L.J.Z.), blinded for clinical data, independently scored the slides in at least five fields using a 40× objective.

### Flow cytometry

100 μl of EDTA-anticoagulated peripheral blood was stained with 5 μl of the following fluorochrome-conjugated antibodies: anti-human CD3-APC (BD Pharmingen™ APC Mouse Anti-Human CD3), anti-human CD279-PE (BD Pharmingen™ PE Mouse Anti-Human CD279) and anti-human CD4-FITC (BD Pharmingen™ FITC Mouse Anti-Human CD4) or anti-human CD8-FITC (BD Pharmingen™ FITC Mouse Anti-Human CD8) and incubated for 30 minutes at 4 °C in dark. Next, the cells were lysed by the BD FACS Lysing Solution (Becton Dickinson) for 15 minutes at 4 °C, and washed twice with cold PBS. Isotype control antibodies: anti-human CD3, anti-human CD279 and anti-human CD4 or anti-human CD8 were used for setting compensation and to set gates. Specimen assays were performed using the BD LSRFortessa (BD Biosciences) and the data were analyzed by FlowJo 10.0.7 software.

### Statistical analysis

Statistical analyses and graphical representations were performed using SPSS 23.0 (SPSS Inc.; Chicago, IL, USA) and GraphPad Prism 6 (San Diego, CA) software. The student’s t test was used to analyze the differences in PD-1 expressions on peripheral CD4+ or CD8+ T lymphocytes between patients with PDAC or IPMN and healthy donors. It was also used to analyze the differences in the number of CD8+ cells or PD-1+ CD8+ cells between tumor and adjacent non-tumor tissues. The Wilcoxon test was applied to compare changes in the expressions of PD-1 on peripheral CD8+ T cells before and after surgical operation. The χ2 test was used to analyze the correlations between PD-1 expression and clinicopathologic parameters in patients with PDAC. Survival curves were evaluated using the Kaplan–Meier method and differences between survival curves were tested by the log-rank test. A two-sided P-value < 0.05 was considered statistically significant.

## Electronic supplementary material

Supplementary Table 1
