# Supplementary material for: Prognostic value of programmed cell death protein 1 expression on CD8+ T lymphocytes in pancreatic cancer
Source: Sci Rep. 2017 Aug 10;7:7848. doi: 10.1038/s41598-017-08479-9 (PMC5552822; doi:10.1038/s41598-017-08479-9)
Supplement: Supplementary file 1 — Supplementary Table 1 [file 41598_2017_8479_MOESM1_ESM.doc]

# Prognostic value of programmed cell death protein 1 expression on CD8+ T lymphocytes in pancreatic cancer

Tao Shen, Liangjing Zhou, Hua Shen, Chengfei Shi, Shengnan Jia, GuoPing Ding & Liping Cao

Address: Department of General Surgery, Sir Run Run Shaw Hospital, School of Medicine, Zhejiang University, Hangzhou 310000, China.

Correspondence and requests for materials should be addressed to G.P.D. (email: dinguop@zju.edu.cn) or L.P.C. (email: caolipingzju@126.com)

|  | **PD-1 expression on CD4+ T lymphocytes (Mean±SD%)** | **PD-1 expression on CD8+ T lymphocytes (Mean±SD%)** |
| --- | --- | --- |
| **PDAC** | 46.62±11.94^a,b^ | 51.08±13.22**^d^**,**^e^** |
| **IPMN** | 50.77±8.22^a,c^ | 31.96±12.72**^d^**^,f^ |
| **Health** | 48.95±11.51^b,c^ | 28.04±10.27**^e^**^,f^ |

Supplementary Table 1. Comparisons of PD-1 expression on peripheral CD4+ or CD8+ T lymphocytes between each group. PD-1 expression on CD4+ T lymphocytes: ^a^ PDAC vs. IPMN p=0.055, ^b^ PDAC vs. IPMN p=0.441, ^c^ PDAC vs. IPMN p=0.484; PD-1 expression on CD8+ T lymphocytes: **^d^** PDAC vs. IPMN **p<0.001**, **^e^** PDAC vs. IPMN **p<0.001**, ^f^ PDAC vs. IPMN p=0.236. The bold number represents the P-value with significant differences (p<0.05).
